# Safety and effectiveness evaluation of a two-handed technique combining harmonic scalpel and laparoscopic Peng’s multifunction operative dissector in laparoscopic hemihepatectomy

**DOI:** 10.1186/s12957-021-02311-5

**Published:** 2021-07-04

**Authors:** Jingwei Cai, Guixing Jiang, Yuelong Liang, Yangyang Xie, Junhao Zheng, Xiao Liang

**Affiliations:** Department of Hepatobiliary and Pancreatic Surgery, Sir Run Run Shaw Hospital, Zhejiang University School of Medicine, 3 East Qingchun Road, Hangzhou, 310016 People’s Republic of China

**Keywords:** Laparoscopic hemihepatectomy, Harmonic scalpel, Laparoscopic Peng’s multifunction operation dissector, Two-handed technique

## Abstract

**Objectives:**

This study was designed to evaluate the safety and effectiveness of a two-hand technique combining harmonic scalpel (HS) and laparoscopic Peng’s multifunction operative dissector (LPMOD) in patients who underwent laparoscopic hemihepatectomy (LHH).

**Methods:**

We designed and conducted a case-control study nested in a prospectively collected laparoscopic liver surgery database. Patients who underwent LHH for liver parenchyma transection using HS + LPMOD were defined as cases (*n* = 98) and LPMOD only as controls (*n* = 47) from January 2016 to May 2018. Propensity score matching (1:1) between the case and control groups was used in the analyses.

**Results:**

The case group had significantly less intraoperative blood loss in milliliters (169.4 ± 133.5 vs. 221.5 ± 176.3, *P* = 0.03) and shorter operative time in minutes (210.5 ± 56.1 vs. 265.7 ± 67.1, *P* = 0.02) comparing to the control group. The conversion to laparotomy, postoperative hospital stay, resection margin, the mean peak level of postoperative liver function parameters, bile leakage rate, and others were comparable between the two groups. There was no perioperative mortality.

**Conclusions:**

We demonstrated that the two-handed technique combing HS and LPMOD in LHH is safe and effective which is associated with shorter operative time and less intraoperative blood loss compared with LPMOD alone. The technique facilitates laparoscopic liver resection and is recommended for use.

## Introduction

Massive bleeding is a big challenge for hepatic resection, especially hemihepatectomy. It is a cause of death during the surgery and hemorrhage after the surgery and affects prognosis as well. Increased intraoperative bleeding during liver surgery has been reported to have a negative impact on postoperative recovery and prognosis [1]. Massive blood loss during liver surgery is related to the high risk of postoperative mortality and recurrence of hepatocellular carcinoma. Massive intraoperative blood loss may lead to a longer time of systemic hypoperfusion and affects oxygen delivery to vital organs [2]. In addition, perioperative blood transfusion due to massive blood loss is associated with worse survival outcomes in postoperative patients [3, 4]. Therefore, techniques which can help minimize bleeding during the hepatic resection are demanded to be developed.

Over the last two decades, laparoscopic surgery has been employed in various surgical fields [5–9]. With the development of laparoscopic techniques, laparoscopic liver resection has widely been carried out with the feasibility, safety, oncological efficiency, and surgical indications [10, 11]. Laparoscopic hemihepatectomy (LHH) was associated with less intraoperative blood loss, better postoperative recovery, and a shorter length of hospital stay. Safe and effective parenchyma transection is a critical step in LHH, which is dependent on the efficient management of the parenchyma division and hemostasis. Previous studies have compared the clinical benefits of different methods of hepatic transection in open hepatectomy [12, 13]. No standardized or best method, however, has been proposed for LHH to date and the appropriate laparoscopic surgical instruments for transecting the liver parenchyma are still controversial. The objective of this study was to report a novel simple method to perform laparoscopic hemihepatectomy and to explore the feasibility of this new technique.

## Methods

### Study design and patients

A retrospective case-control study nested in a cohort study was conducted using prospectively collected data from medical records system in the Sir Run Run Shaw Hospital at Zhejiang University School of Medicine from January 2016 to May 2018. All data were entered into the database by Dr. JHZ. All patients were given and signed the consent form. Patients who underwent LHH during the study period by two-handed liver surgery technique HS + LPMOD were defined as cases and LPMOD only as controls. Patients who received the liver wedge resection, liver segment resection, and extended hemihepatectomy were excluded from this study. In total, 497 patients were excluded, and 145 patients were included in the study. To be more specific, 497 cases were excluded due to unclear hepatectomy type (*n* = 32), missing hepatectomy type (*n* = 10), partial hepatectomy (*n* = 330), and 125 segmental hepatectomy (*n* = 125). Of the 145 patients included in the study, 98 patients underwent the liver parenchyma transection HS plus LPMOD method while 47 underwent LPMOD only.

### Surgical procedures

**Fig. 1:**
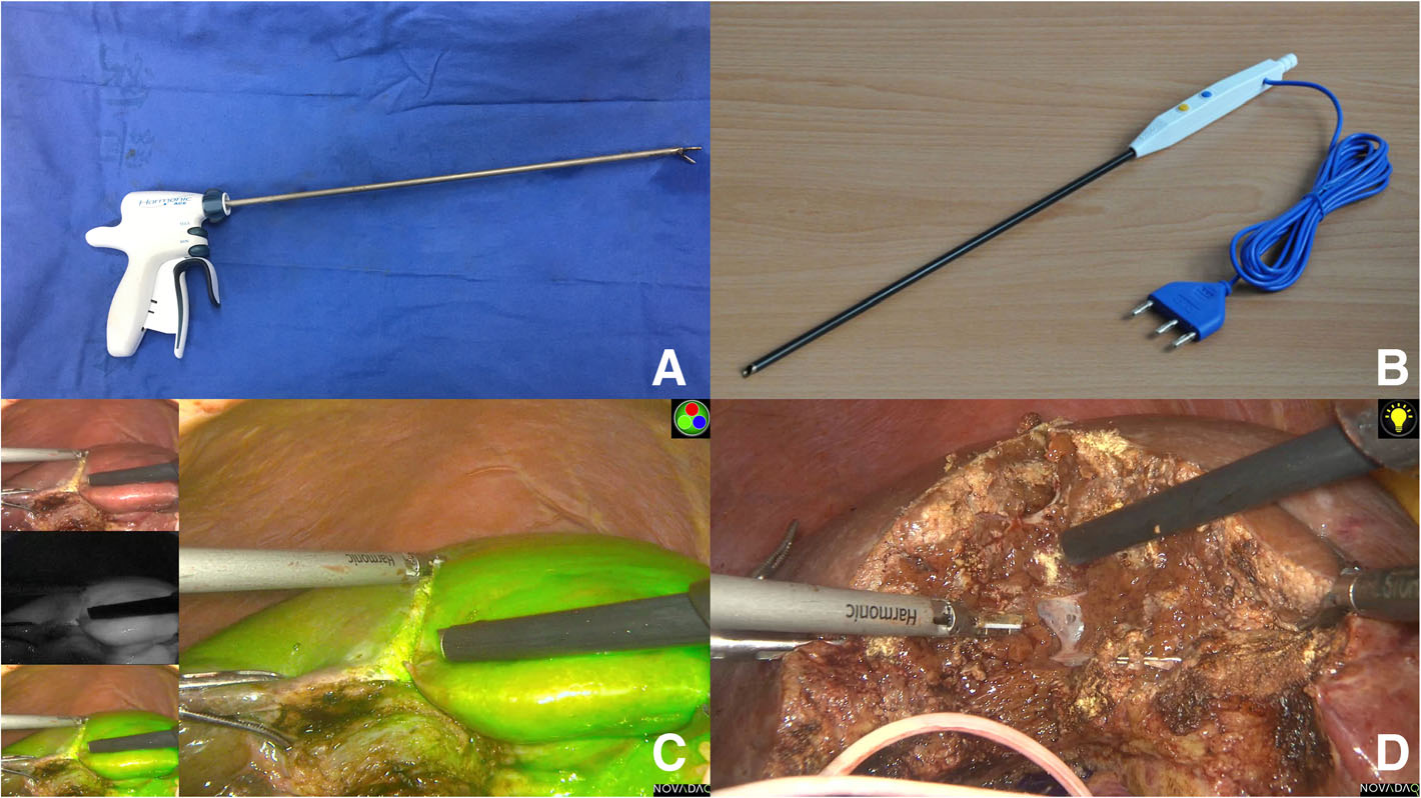
Some critical surgical instruments used in laparoscopic hemihepatectomy (LHH). **A** Harmonic scalpel (HS). **B** Laparoscopic Peng’s multifunction operative dissector (LPMOD). **C** Liver parenchyma transection was started with HS. **D** Liver parenchyma is crushed and divided by LPMOD combined with HS and intrahepatic ducts and vessels were observed

Several critical surgical instruments used in LHH were shown in Fig. 1. Patients should be placed in the supine position. Three surgeons including one primary surgeon and two assistants were needed. The primary surgeon stood on the left side of the patient. Carbon dioxide pneumoperitoneum was set at 10–14 mmHg. LHH was routinely performed with a four-port method. The observation port was placed above the umbilicus (10 mm); the main operating port was put below the xiphoid process (12 mm) and on the right collarbone midline (12 mm); and the assistant port was put on the right axillary frontline (5 mm).

Following routine laparoscopic exploration, intraoperative sonography was used to confirm the extent of diseases and the relationship of important vessels and guide the appropriate parenchyma transection plane. Hepatoduodenal ligament occlusion was performed routinely by the Pringle maneuver to occlude the hepatic inflow for 10 min and released for 5 min [14].

In the HS + LPMOD group, the primary surgeon needed to operate with both hands: the left hand held the HS, and the right hand held LPMOD to dissect the parenchyma. The HS was applied to crack liver capsule approximately 2 cm away from the superficial liver tissues, while the LPMOD was simultaneously used to expose, electrically coagulate, and suction. Alternated operation of two hands was performed to cut and crush the liver parenchyma in different parts of the transection plane. Vessels less than 3 mm were directly sealed by HS. Liver parenchyma hemorrhage was managed by LPMOD.

In the LPMOD only group, the technique for LHH was described previously in our institution [15]. Briefly, liver parenchyma is crushed and aspirated, and then intrahepatic ducts and vessels can be dissected and preserved safely for clipping or ligation. Vessels less than 3 mm would be electrically coagulated by LPMOD.

In both groups, the vessels larger than 3 mm were cut by HS after clipping by Hem-lock or titanium clap on the remnant side. Laparoscopic vascular stapler was adopted to divide hepatic pedicles or some hepatic veins which were larger than 10 mm.

Evaluated endpoints were operative outcomes including the type of liver resection, intraoperative blood loss, blood transfusion, operative time, conversion to laparotomy, postoperative hospital days, hospital mortality, resection margin, postoperative bile leakage, and postoperative liver function. The evaluation of postoperative liver function was assessed by measurements of postoperative mean peak level of alanine aminotransferase (ALT), aspartate transaminase (AST), total bilirubin (TBIL), and prothrombin time (PT). Biliary fistula was defined as bilious drainage lasting more than 7 days after the surgery [16].

### Statistical analysis

All analyses were performed using the SPSS 22.0 statistical software. Continuous variables were expressed by mean and standard deviation (SD), and categorical variables were expressed by number and percentage for each category. Paired-sample t test or Wilcoxon rank-sum test was used for continuous variables. And Pearson chi-square test or Fisher’s exact test was used for categorical data. To eliminate some potential biases due to confounding factors, the propensity score matching (PSM) technique was used. A matching ratio of 1 to 1 was used based on the “nearest neighbor” method [17]. The propensity scores were estimated using logistic regression which included the following variables: age, sex, body mass index (BMI), American Society of Anesthesiologists (ASA) score, level of serum alpha-fetoprotein (AFP), liver cirrhosis, and tumor characteristics (number and size). After 1 to 1 matching, 47 patients were included in the analyses in each group which had similar baseline and pathological characteristics. *P* value < 0.05 was considered as statistically significant.

## Results

Baseline characteristics of the case (HS + LPMOD) and control (LPMOD only) group are presented in Table 1. The cases comprised 54 patients with a histopathologically confirmed malignant tumor, while 25 patients with malignant hepatic tumors in the control group. There was not a significant difference between two groups in terms of the distribution of sex, histopathologic malignancy, ASA score, AFP, liver cirrhosis, and common pathological characteristics. Significant differences were observed depending on the group; the case group was more likely to have a lower BMI (*P* = 0.02) and higher elderly population (*P* = 0.03) than the control group. After PSM analysis, however, two groups had similar clinicopathologic features.

**Table 1:** Demographic and pathologic factors before and after propensity score matching among the patients in the study

	Entire cohort			Propensity-matched cohort		
	HS + LPMOD (*n* = 98)	LPMOD (*n* = 47)	*P* value	HS + LPMOD (*n* = 47)	LPMOD (*n* = 47)	*P* value
Age, mean (SD)	58.6 (11.1)	55.7 (11.8)	0.03*	56.4 (10.7)	55.7 (11.8)	0.36
Sex			0.20			0.41
Male, n (%)	65 (66.3)	26 (55.3)		22 (46.8)	26 (55.3)	
Female, n (%)	33 (33.7)	21 (44.7)		25 (53.2)	21 (44.7)	
BMI, mean (SD)	21.1 (2.2)	22.7 (2.4)	0.02*	21.8(1.9)	22.7 (2.4)	0.34
ASA, n (%)			0.99			0.90
I	28 (28.6)	13 (27.7)		15 (31.9)	13 (27.7)	
II	60 (61.2)	29 (61.7)		27 (57.4)	29 (61.7)	
III	10 (10.2)	5 (10.6)		5 (10.6)	5 (10.6)	
AFP, n (%)			0.23			0.81
Increased (≥ 400 ng/mL)	15 (15.3)	11 (23.4)		12 (25.5)	11 (23.4)	
Not increased (< 400 ng/mL)	83 (84.7)	36 (76.6)		35 (74.5)	36 (76.6)	
Histopathologic diagnosis			0.89			0.95
Colorectal carcinoma	14 (14.3)	5 (10.6)		5 (10.6)	5 (10.6)	
Hepatocellular Carcinoma	36 (36.7)	19 (40.4)		20 (42.6)	19 (40.4)	
Cholangiocarcinoma	4 (4.1)	1 (2.1)		2 (4.3)	1 (2.1)	
Benign	38 (38.8)	20 (42.6)		19 (40.4)	20 (42.6)	
Other	6 (6.1)	2 (4.3)		1 (2.1)	2 (4.3)	
Cirrhosis, n (%)	16 (16.3)	10 (21.3)	0.47	12 (23.4)	10 (21.2)	0.63
Pathologic characteristic						
Number of tumors, n (SD)	1.5 (0.3)	1.2 (0.5)	0.32	1.3 (0.3)	1.2 (0.5)	0.54
Largest tumor size, cm (SD)	6.1 (3.5)	5.8 (2.8)	0.46	6.1 (3.3)	5.8 (2.8)	0.62

*Abbreviations*: *AFP* alpha-fetoprotein, *ASA* American Society of Anesthesiologists, *BMI* body mass index, *SD* standard deviation. *HS* harmonic scalpel, *LPMOD* laparoscopic Peng’s multifunction operative dissector

*P<0.05; **P<0.01 between two groups

In the overall cohort, the intraoperative blood loss was significantly diminished in the case group compared with the control group (mean, 158.5 vs. 221.5 ml, *P* < 0.01). In addition, the HS + LPMOD group had a significantly shorter operative time in minutes than that in the LPMOD only group (mean, 202.4 vs. 265.7 min, *P* < 0.01). In the PSM cohort, mean operative times were shorter in the HS + LPMOD group (mean, 210.5 vs. 265.7 min, *P* = 0.02), and the HS + LPMOD group experienced less intraoperative blood loss compared to the control group (mean, 169.4 vs. 221.5 ml, *P* = 0.03). There were no differences in blood transfusion rate, conversion to laparotomy, postoperative hospital stay, resection margin, and perioperative hospital mortality between the two groups either before or after PSM (Table 2).

**Table 2:** Operative factors before and after propensity score matching among the patients in the study

	Entire cohort			Propensity-matched cohort		
	HS + LPMOD (*n* = 98)	LPMOD (*n* = 47)	*P* value	HS + LPMOD (*n* = 47)	LPMOD (*n* = 47)	*P* value
Hemihepatectomy			0.58			0.68
Left (%)	59 (60.2)	21 (44.7)		28 (59.6)	26 (55.3)	
Right (%)	39 (39.8)	21 (55.3)		19 (40.4)	21 (44.7)	
Blood loss, mL (SD)	158.5 (124.2)	221.5 (176.3)	<0.01**	169.4 (133.5)	221.5 (176.3)	0.03*
Blood transfusion, n (%)	26 (26.5)	13 (27.7)	0.72	14 (29.8)	13 (27.7)	0.82
Operative time, minutes (SD)	202.4 (47.5)	265.7 (67.1)	<0.01**	210.5 (56.1)	265.7 (67.1)	0.02*
Conversion to laparotomy**,** n (%)	5 (5.1)	3 (6.4)	0.75	4 (8.5)	3 (6.4)	0.69
Postoperative hospital stay, days (SD)	14.1 (9.4)	13.7 (9.4)	0.80	14.0 (8.7)	13.7 (9.4)	0.83
Hospital mortality, n (%)	0	0	–	0	0	–
Resection margin$			0.97			1.00
R0 (%)	96 (98.0)	46 (97.9)		46 (97.9)	46 (97.9)	
R1 (%)	2 (2.0)	1 (2.1)		1 (2.1)	1 (2.1)	
R2	0	0		0	0	

*Abbreviations*: *HS* harmonic scalpel, *LPMOD* laparoscopic Peng’s multifunction operative dissector, *SD* standard deviation

$ Resection margin: R0: no microscopically identifiable tumor remnants, R1: microscopically identifiable tumor remnants R2: macroscopically identifiable tumor remnants

*P<0.05; **P<0.01 between two groups

The postoperative parameters of liver function increased dramatically postoperatively, but no significant differences were observed in the mean peak level of ALT, AST, TBIL, and PT between the two groups either before or after PSM (Table 3). The bile leakage rate was comparable between the two groups (7.1% vs. 8.5%, *P* = 0.77; 6.4% vs. 8.5%, *P* = 0.69, respectively) before and after matching. The bile fistula was resolved following a short course of drainage and antibiotic therapy in both groups.

**Table 3:** Postoperative variables before and after propensity score matching

	Entire cohort			Propensity-matched cohort		
	HS + LPMOD (*n* = 98)	LPMOD (*n* = 47)	*P* value	HS + LPMOD (*n* = 47)	LPMOD (*n* = 47)	*P* value
Bile leakage n (%)	7 (7.1)	4 (8.5)	0.77	3 (6.4)	4 (8.5)	0.694
Liver functions						
Mean peak AST (± SD)	253.8 (146.4)	316.7 (187.8)	0.33	260.8 (156.5)	316.7 (187.8)	0.431
Mean peak ALT (± SD)	237.2 (126.1)	280.7 (223.6)	0.21	242.0 (143.2)	280.7 (223.6)	0.232
Mean peak total bilirubin (± SD)	27.0 (4.7)	29.7 (5.5)	0.65	27.3 (3.6)	29.7 (5.5)	0.703
Mean peak prothrombin time (± SD)	15.3 (3.4)	15.1 (2.5)	0.24	15.2 (3.1)	15.1 (2.5)	0.262

*HS* harmonic scalpel, *LPMOD* laparoscopic Peng’s multifunction operative dissector, *ALT* alanine transaminase, *AST* aspartate transaminase

## Discussions

Our study found that our two-handed technique combining HS and LPMOD in LHH can significantly reduce intraoperative blood loss and shorten operative time, which is of great clinical value. The proposed benefits of this novel laparoscopic techniques over traditional one-hand hepatectomy techniques are numerous including quicker physical recovery and decreased postoperative pain and general preference by patients. Synergistic combinations of the advantages of different hepatectomy tools instead of individual hepatectomy technique per se not only optimize the surgical procedures but improve the safety and effectiveness of challenging hemihepatectomy through reducing intraoperative blood loss and shortening operative time which substantively increase the survival rate with better prognosis of patients with liver diseases, especially cancer patients.

In 2005, Aloia et al. [18] first reported a two-surgeon technique; two surgeons were allowed to employ two instruments to participate in the parenchymal transection for major hepatectomies. The primary surgeon dissected the liver parenchyma by ultrasonic dissection (UD) device from the left side of the patient. Simultaneously, the assistant surgeon held the saline-linked cautery (SLC) device that stood next to the patient’s right side. Aloia showed that the “two-surgeon technique,” using a combination of UD and SLC in hepatic resection resulted in shorter operative time and a reduction in the duration of hepatoduodenal ligament occlusion, while the postoperative liver function and complications rate were similar to that of the “one-surgeon technique” group. Mitsuhisa Takatsuki [19] claimed that the blood loss and donor complications in living donor hepatectomies significantly reduced when using the two-surgeon technique with Cavitron Ultrasonic Surgical Aspirator (CUSA) and SLC, while the early graft function and postoperative recipient survival did not differ between the two groups. This finding might be explained by three reasons: (a) the “two-surgeon technique” provides surgeons with less time for exchanging surgical instruments for dissection and coagulation leading to the acceleration of the operation process and (b) the interactive participation of two surgeons during the parenchyma transection promoted the efficiency of surgical processes [20].

Accurate hemostasis is the key achievement in successful laparoscopic liver resection. It also depends on a tacit cooperation between the primary surgeon and assistants. Recent studies have investigated the two-surgeon technique to optimize the surgical procedure of LHH and indicated that it can notably ameliorate the efficiency of liver transection, shorten the operative time, and reduce intraoperative blood loss [18–20]. Nevertheless, cooperation between surgeons is essential to optimize the management of surgical procedures, which is always difficult. The learning curve is steep and the assistant surgeons demand a relatively long training period.

Inspired by the two-surgeon technique, our well-practiced surgeon team started applying our two-handed technique, which combined LPMOD and HS to complete laparoscopic liver parenchyma transection. The operator could manipulate both the LPMOD and HS in real-time by himself to dissect the liver parenchyma in different parts of the transection plane. The two-handed technique can achieve the similar effect with the traditional hepatectomy method. Moreover, the accidental hemorrhage can be managed more accurately and immediately while the surgeon is well trained with both hands. The instructions for the two-handed operation issued by the primary surgeon were more precise and would not cause any misunderstanding which may occur among surgeons when performing the two-surgeon technique. The advantages of two-handed technique can be absolutely utilized by the surgeon during the operation without exchanging or passing instruments frequently. The improvement accelerates the surgical process significantly and optimizes the quality of operation. However, this technique is accompanied by difficulties including adaptation of the non-dominant hand, so intensive trainings are usually required to master the technique. Meanwhile, the role of assistant surgeons cannot be neglected since they play critical roles in the field of vision exposure. The assisting surgeon can still use their free hand to employ an instrument to aid the surgery.

To our knowledge, this is the first study to evaluate the safety and effectiveness of the two-handed technique combined with HS and LPMOD for hepatic parenchyma transection in LHH. With similar baseline demographics and tumor characteristics, the two-handed technique significantly reduced blood loss and shortened operative time compared with the control group. Variables analyzed including blood transfusion, conversion to laparotomy, and the length of postoperative hospital stay and resection margin were comparable between the two groups. No significant differences were observed in postoperative liver function parameters between the two groups.

Hemorrhage during laparoscopic hemihepatectomy is mainly due to damage of the hepatic vein or branches inside the liver parenchyma [21]. Our two-handed technique can manage accidental hemorrhage perfectly since this technique combined the superiority of small vessels sealing function of HS with coordinating manipulation of LPMOD which is effective for hemostasis. It has been shown that massive blood loss is associated with an increased risk of death and recurrence after radical resection since serious intraoperative bleeding may facilitate dissemination of tumors, which could result in an increased risk of local recurrence [22–24].

Our study revealed that the two-handed technique group showed significantly shorter operative times than the classic technique group. This might be explained by the fact that in the “one hand” group, dissection of the vessels during liver parenchyma transection requires a supplementary procedure (harmonic scalpel dissection after clipping on the remnant side) to control bleeding at the sectioning plane, which cost extra time. However, with the help of the two-handed technique, HS can seal and occlude the small vessel simultaneously when ducts and vessels were exposed by LPMOD. Therefore, ultrasonic scissors that are combined with LPMOD could be shown to shorten the operation time and ameliorate the safety of vessel ligation.

There was no difference in the mean peak level of postoperative liver function measured by ALT, AST, TBIL, and PT between the two groups. The complication of bile leakage during hemihepatectomy may be affected by the tools chosen for parenchyma transection. In the two-handed technique group, bile leakage occurred in 3 patients (6.4%) who were followed to be treated by long-time drainage without other invasive interventions. The incidence of bile leakage was comparable between the two groups (6.4% vs. 8.5%) after PSM. Besides, there was no perioperative death in both groups. Therefore, our data suggested that the novel surgical techniques can also be safely and effectively performed in LHH.

Our study is limited in several aspects. Firstly, this is a case-control study, so some potential selection bias might be concerned but this bias was minimized by applying PSM in the analyses and or controlled by that the study is nested in a prospectively collected database. Secondly, only patients who underwent LHH were included in this study so that our technique may not be generalized to other types of liver resection such as wedge resection and anatomical segmentectomy that were excluded in this study. Thirdly, surgeons are required to take some time to get trained and practiced if they want to use this technique. Future studies would need to assess the safety and effectiveness of the two-handed technique in other types of liver resection.

In conclusion, this study shows the new two-handed minimally invasive approach combining HS and LPMOD together provided patients with a safer and more effective technique of hepatic parenchymal transaction. Although no single technique is available to effectively complete the division and hemostasis during laparoscopic liver parenchyma transection, we can utilize the strengths of conceptually different hepatic transection instruments together to create a new operative technique like our two-handed technique. This technique can synergize the advantages of different hepatectomy tools, improve hepatic transection efficiency, optimize surgical procedures, reduce intraoperative blood loss, and shorten operative time. We strongly recommend this emerging technique to be applied in other laparoscopic hepatectomy institutions.

## Data Availability

All the data can be obtained from the author by email (12018274@zju.edu.cn).

## Abbreviations

HS: Harmonic scalpel
LPMOD: Laparoscopic Peng’s multifunction operative dissector
LHH: Laparoscopic hemihepatectomy
ALT: Alanine aminotransferase
AST: Aspartate transaminase
TBIL: Total bilirubin
PT: Prothrombin time
PSM: Propensity score matching
BMI: Body mass index
AFP: Alpha-fetoprotein
